# Antimicrobial and Synergistic Activity of Metformin Against *Helicobacter pylori*

**DOI:** 10.1155/cjid/3542284

**Published:** 2026-05-12

**Authors:** Walaa Hashem Rajab, Nidal Jaradat, Qusay Abdo, Nour Aljouda, Mohammad Qadi

**Affiliations:** ^1^ Department of Medical and Health Sciences, Faculty of Graduate Studies, An-Najah National University, Nablus, State of Palestine, najah.edu; ^2^ Department of Medical Technology, Faculty of Allied Medical Sciences, Arab American University Palestine, Jenin, State of Palestine, aaup.edu; ^3^ Faculty of Pharmacy, An-Najah National University, Nablus, State of Palestine, najah.edu; ^4^ Department of Gastroenterology, An-Najah National University Hospital, Nablus, State of Palestine, najah.edu; ^5^ Department of Medicine, Faculty of Medicine and Allied Medical Sciences, An-Najah National University, Nablus, State of Palestine, najah.edu; ^6^ Department of Biomedical Sciences and Basic Clinical Skills, Faculty of Medicine and Allied Medical Sciences, An-Najah National University, Nablus, State of Palestine, najah.edu

**Keywords:** antibiotic combination, checkerboard, *Helicobacter pylori*, MBC, metformin, MIC

## Abstract

**Introduction:**

*Helicobacter pylori* (*H. pylori*) infection is highly prevalent worldwide and is associated with multiple gastroduodenal pathologies, including gastric cancer. The increasing pattern of drug resistance among *H. pylori* limits available treatment options. To overcome this challenge, drug repurposing and the use of current medications as adjuvant therapies have emerged as a valuable alternative strategy. Metformin is a commonly prescribed antidiabetic drug for Type 2 diabetes mellitus (T2DM); it has been shown to have antibacterial properties. This study aimed to investigate the anti‐*H. pylori* activity of metformin and evaluate its potential to enhance the efficacy of six conventional antibiotics used to treat *H. pylori* infections.

**Methods:**

The antibacterial effect of metformin on *H. pylori* was evaluated using both clinical and reference laboratory strains by detecting the minimum inhibitory concentration (MIC) and minimum bactericidal concentration (MBC). Furthermore, a checkerboard assay was conducted to assess the combined effect of metformin with six conventional antibiotics used to treat *H. pylori* infection.

**Results:**

Metformin showed limited antibacterial activity against *H. pylori,* with MIC and MBC values ranging between 6.83 and 9.75 mM (1131–1615 μg/mL). MIC/MBC ratios and time‐kill study suggested a bactericidal effect against the tested *H. pylori* strains. The fractional inhibitory concentration index (FICI) findings for the tested combinations were 0.52–1.0, including the MDR strain, indicating an additive effect. No synergistic or antagonistic interactions were observed.

**Conclusion:**

The additive effect and the bactericidal activity of metformin suggest a potential clinical relevance for diabetic patients already receiving this medication. However, comprehensive studies are needed to further evaluate safety, efficacy, and the underlying killing mechanism of metformin against *H. pylori*.

## 1. Introduction

*Helicobacter pylori* (*H. pylori*) is a Gram‐negative, spiral‐shaped bacterium [1]. It is one of the most prevalent infectious agents, affecting more than half of the global population [2]. *H. pylori* is the primary cause behind several gastroduodenal disorders, including chronic gastritis, peptic ulcers, and gastric adenocarcinoma that may develop in approximately 3% of untreated *H. pylori* infections [3, 4].

Given its high prevalence, the classification by the World Health Organization (WHO) as a Group 1 definite carcinogen [5], and the lack of an effective and safe vaccine [6], there is growing interest in identifying novel and effective treatment strategies [7]. Adjuvant use to enhance the potency of existing antibiotics represents a viable therapeutic approach. This favorable approach uses non‐antibiotic drugs with antibacterial activities to potentiate antibiotic performance [8]. Such innovative therapeutic strategies may improve therapeutic success while limiting the rise of antimicrobial resistance, pathogenicity, and virulence [9].

Metformin, chemically known as 1,1‐dimethylbiguanide, is the most prescribed antihyperglycemic agent. It is recommended by the American Diabetes Association (ADA) as the initial therapy for managing Type 2 diabetes mellitus (T2DM) [10]. Metformin is considered safe, with a relatively low rate of adverse effects. Growing evidence has emphasized its potential antibacterial activity. Recent studies illustrated not only its direct antibacterial activity but also its role in modulating the gut microbiome [11, 12]. Emerging data from in vivo and in vitro studies observed metformin’s antibacterial properties against antibiotic‐resistant and susceptible bacterial isolates. For instance, Masadeh et al. described metformin as a potential adjuvant against drug‐resistant bacteria such as methicillin‐resistant *Staphylococcus aureus* (MRSA) and *Pseudomonas* spp. [13]. Metformin has also been shown to reverse tetracycline resistance among a group of drug‐resistant bacteria [14, 15]. Moreover, a group of researchers from Taiwan pointed out that the use of metformin among T2DM patients is associated with a considerably lower incidence of *H. pylori* infection in a dose‐dependent manner [16]. According to our knowledge, limited studies have investigated metformin’s activity against *H. pylori* [17]. Courtois et al. reported its direct effect on *H. pylori*: through in vitro and in vivo studies, metformin at concentrations of 2–10 mM significantly reduced *H. pylori* count in a dose‐dependent manner. Additionally, the disk diffusion agar assay reported inhibition zone diameters of 11–24 mm with 0.5 M metformin and 21–44 mm with 1 M, compared to no activity using 0.1 M. Furthermore, the same team reported a significantly lower number of gastric *H. pylori* among metformin–treated mice (10 mg/mouse/day) compared to untreated infected controls [17].

Reports describing the underlying mechanism for metformin’s antibacterial activity against *H. pylori* are limited. However, a recent study reported a potential mediated by a downregulated *cagA* gene expression—a potent virulence factor associated with gastric ulcer—suggesting that metformin may exert antivirulence effects. They also found that metformin reduced *H. pylori*‐triggered inflammation via increasing Treg cells [18]. Although the exact mechanism against *H. pylori* remains unclear, the current literature has proposed several pathways to explain metformin’s antibacterial activity in specific bacterial species. These include the activation of adenosine monophosphate‐activated protein kinase (AMPK), potential production of mitochondrial reactive oxygen species (ROS) in *Mycobacterium tuberculosis* [19], and inhibition of virulence through suppression of biofilm formation in various Gram‐negative bacteria, including *Pseudomonas aeruginosa, Acinetobacter baumannii, Klebsiella pneumoniae,* and *Escherichia coli* [14, 20–22], reduced urease production by *K. pneumoniae* [21], and disruption of bacterial plasma membranes [14]. However, those mechanisms remain hypothetical and require further investigation.

Researchers devote enormous attention to screen synergy interactions, tackling the crisis of emergent antibiotic‐resistant strains, including the multidrug resistant (MDR). Thus, the synergy activity identifies drug combinations that exhibit enhanced antibacterial activity over using each agent separately [23]. The checkerboard assay represents an established in vitro technique for investigating the antibacterial properties of novel drug combinations. This two‐dimensional dilutional assay utilizes 2‐fold dilution of the first drug across the rows while the second one undergoes 2‐fold serial dilution across the columns. The outcome of the combination could be evaluated based on fractional inhibitory concentration index (FICI) mathematics [24].

Moreover, for *H. pylori*, insufficient data confirm the antibacterial and synergistic activity of metformin–antibiotic combinations. A recent study investigated the effect of combining metformin with amoxicillin on a single *H. pylori* strain [18]. However, there are no available reports examining the effect of combining metformin with other commonly used antibiotics for *H. pylori* treatment. Therefore, the current study aimed to evaluate the antibacterial activity of metformin against *H*. *pylori* and to explore its potential synergistic effects when combined with the standard antibiotics, using the checkerboard assay and FICI. Our study explored the antibacterial activity through endpoint standardized assays, including minimal inhibitory concentration (MIC) and minimal bactericidal concentration (MBC) in addition to the dynamic assay time‐kill protocol that permits investigating the speed of killing over time, evaluating pharmacodynamics to differentiate time‐dependent from dose‐dependent killing, and identifying any possible regrowth during the incubation period. Here, we used reference and clinical strains with variable resistance profiles to assess the role of metformin in boosting antibiotic effectiveness against *H. pylori*.

## 2. Methodology

### 2.1. *H. pylori* Strains and Growth Conditions

#### 2.1.1. Reference Strains

Two reference strains of *H. pylori* were used in this study: ATCC 43504 (a metronidazole‐resistant strain and susceptible to the remaining studied antibiotics, used as a quality control strain in the MIC assay) and the ATCC 700824 strain/J99 (susceptible to all tested antibiotics and positive for CagA and VacA virulence factors). These strains were obtained from the American Type Culture Collection (ATCC) in Manassas, Virginia, United States.

#### 2.1.2. Clinical Strain Isolation

Gastric biopsies from patients undergoing routine gastroscopic procedures at An‐Najah National University Hospital (NNUH) were collected between 1 May 2024 and 31 August 2024 to isolate *H. pylori* clinical strains. Three *H. pylori* clinical strains were selected with variable antibiotic resistance profiles (designated S1, S2, and S3). Patients’ inclusion criteria were (1) ability and agreement to participate in the study and to provide a written consent and (2) a positive rapid urease test to confirm *H. pylori* infection (Strong Biotech Helicotec UT Plus, CR Kennedy, Australia). Patients who had taken antibiotics within the previous 4 weeks or were under 18 years old were excluded.

Biopsies were taken from both the antrum and corpus, placed in sterile cryotubes with 300 μL brain heart infusion (BHI) broth and 10% fetal bovine serum (FBS) (Oxoid, Hampshire, UK), and transported at room temperature [25]. Samples were homogenized within two hours using a portable ultrasonic homogenizer (MRC Laboratory Instrument HOG‐160‐1, 5 mm generator [18900113], UK) for 30 s; if delayed beyond 4 h, they were refrigerated at 4°C for up to 24 h.

#### 2.1.3. Culture Media and Growth Conditions

A volume of 100 μL of the homogenized sample was inoculated into Columbia blood agar base (Oxoid, Hampshire, UK) supplemented with Dent antibiotic (Oxoid, Hampshire, UK) and 7% (v/v) lysed horse blood (Oxoid, Hampshire, UK). Dent is a selective supplement for *H. pylori,* added to the medium to reach a final concentration of 10 mg/L vancomycin, 5 mg/L trimethoprim, 5 mg/L cefsulodin, and 5 mg/L amphotericin B. To enhance the selectivity of the Columbia blood agar base, 2 mL of sterile distilled water was mixed with the commercial Dent supplement vial, then filter‐sterilized and added to 500 mL of the agar medium. Plates were incubated at 37°C under microaerophilic conditions generated by CampyGen Sachets 2.5 L (Oxoid, Hampshire, UK) for 7–10 days. Isolates were identified by their colony appearance (tiny colorless), Gram staining (negative), morphology (microscopic spiral shape), and positive urease, oxidase, and catalase tests.

All isolates were preserved in a mixture of 65% BHI, 25% glycerol, and 10% FBS and stored at −80°C for further use [26]. Molecular confirmation was done using PCR targeting the *H. pylori* nuclease gene (*Nuc*) with the following primers (annealing temperature: 63°C):

• Forward: 5′‐GGC​TGT​TTA​GGG​GTT​TTG​CAA​GC‐3′
• Reverse: 5′‐GCT​ACT​TTT​TGG​TGC​ATG​ATG​CCG‐3′

### 2.2. Preparation of Metformin and Antibiotics

Antibiotics were selected following the European Committee on Antimicrobial Susceptibility Testing (EUCAST) guidelines Version 16.0 [27]. Antibiotics and metformin hydrochloride (PHR1084) were purchased from Sigma Aldrich. The molecular weight of metformin is 165.62 g/mol. Stock solutions of antibiotics were prepared and preserved in aliquots at −80°C. Antibiotics, including levofloxacin (28266), metronidazole (M3761), and tetracycline hydrochloride (T8032), were prepared using pure water, while amoxicillin trihydrate (1031503), rifampicin (R3501), and clarithromycin (Y0000320) were dissolved in dimethyl sulfoxide (DMSO). DMSO concentration was kept below levels known to affect bacterial growth. Working solutions of antibiotics were freshly prepared in Muller–Hinton broth (MHB) supplemented with 10% FBS. Metformin stock (2 M) was similarly prepared in the same medium, and all stock solutions were filtered before use.

### 2.3. MIC

MICs values of metformin and the antibiotics were determined using a standard broth microdilution method as previously described with slight modifications [28, 29]. Clinical breakpoints and assay protocols were performed as per the EUCAST guidelines Version 16.0 [27]. Metformin stock (2 M) and working solutions were freshly prepared and used. Metformin was tested across concentrations from 1 M to 1.95 mM (pH 7.6). Concentrations of the studied antibiotics were amoxicillin trihydrate (0.78–0.0015 μg/mL), tetracycline (1.91–0.0037 μg/mL), clarithromycin (50–0.03 μg/mL), rifampicin (5–0.03 μg/mL), levofloxacin (25–0.048 μg/mL), and metronidazole (250–0.122 μg/mL). Briefly, flat‐bottom 96‐well microplates (Falcon, Cat #351172) were used in the broth microdilution assay. MHB supplemented with 10% FBS was used as the assay medium and for adjusting bacterial density. The *H. pylori* (ATCC 43504) strain was used as quality control during MIC testing. The bacterial inoculum was prepared from 3‐day‐old *H. pylori* colonies grown on Columbia agar base supplemented with 7% lysed horse blood agar (dent‐free). *H. pylori* colonies were suspended in normal saline and adjusted to an optical density of 0.1 at 600 nm using a spectrophotometer (EMC‐16PC‐UV Spectrophotometer). The inoculum was diluted to a final concentration of 5 × 10^5^ CFU/mL and verified by plating into Columbia agar base (dent‐free). For the assay, 200 μL of the test compounds (metformin or relevant antibiotic) were added to wells in column 1 (rows A–F) of a 96‐well microplate. Then, each well received 100 μL of assay medium in columns 2–12 (A–F). Using the multichannel pipette, 2‐fold serial dilutions were performed from column 1 to column 10. Finally, 100 μL of *H. pylori* suspension was added horizontally to wells in columns (1–11), where column 11 served as the positive growth control. Column 12 contained only the assay medium (no bacteria) and served as the negative control.

The plates were incubated for 3 days at 37°C in a microaerophilic condition with gentle shaking at 100 rpm. Following incubation, bacterial growth was assessed visually and validated by measuring absorbance at 600 nm using spectrophotometry. The MIC was defined as the lowest concentration of the treatment that produces no visible turbidity in the relevant well. All MIC assays were performed in triplicate.

### 2.4. MBC

Following the MIC determination, the MBC was conducted as previously described [30]. To determine the MBC, 100 μL from the last turbid well before the MIC, along with 4 wells showing no visible growth (1X to 8X the MIC), was subcultured. A growth control was also spotted in the agar as a purity check. Each sample was spread onto an antibiotic‐free Gonococcal (GC) agar base (M434, Himedia, India) supplemented with 2% hemoglobin [31]. Plates were then incubated at 37°C for 3 days under microaerophilic conditions. Following incubation, plates were examined macroscopically to determine MBC, defined as the minimal concentration that resulted in a 99.9% reduction in bacterial colonies. Reduction of colony count was verified by standard plate counting. All MBC assays were performed in triplicate. The MBC/MIC ratio for metformin was calculated to determine whether its action was bactericidal (< 4) or bacteriostatic (≥ 4) [32].

### 2.5. Killing Kinetics Assay

To evaluate metformin’s killing kinetics for *H. pylori*, a time‐kill study was performed against the reference strain (ATCC 700824), according to Feng et al. with slight modifications [33]. Briefly, in 12‐well plates, bacterial cells (1 × 10^5^ CFU/mL) were inoculated into MHB containing 10% FBS in the presence and absence of metformin, then plates were incubated microaerobically at 37 °C with shaking at 120 rpm for 72 h. *H. pylori* cells were exposed to media (control) or metformin (1X MIC and 2X MIC) for 0, 4, 18, 24, 48, and 72 h. For each sample, 100 μL was aspirated and 10‐fold serially diluted. Subsequently, 100 μL of each dilution was inoculated by a glass spreader onto the surface of Columbia agar base containing 7% laked horse blood; plates were incubated under microaerophilic conditions for 6 days to form isolated colonies. Finally, colonies were counted, and the findings were expressed as the number of Log_10_ (CFU/mL) and presented vs. time. Results are the averages of three independent determinations.

### 2.6. Metformin–Antibiotic Combinations

The potential in vitro dual interactions of metformin with the six selected antibiotics (metronidazole, amoxicillin, rifampicin, tetracycline, levofloxacin, and clarithromycin) were evaluated using a checkerboard broth microtiter assay with minor modifications from previously described protocols [29, 34, 35]. The assay was performed using the two reference *H. pylori* strains (ATCC 43504 and 700824) and three clinical isolates (S1, S2, and S3). The culture media, incubation conditions, and inoculum concentration were consistent with those used in the MIC experiments. Briefly, in a sterile 96‐well microplate, metformin was two‐fold serially diluted along the *Y*‐axis (rows) at concentrations ranging from 2 × MIC to 1/32 × MIC. Antibiotics were likewise diluted twofold across the *X*‐axis (columns), from 2 × MIC to 1/1028 × MIC. Wells were inoculated with 100 μL of *H. pylori* suspension. Wells with dual combinations were generated by mixing 50 μL of the relevant antibiotic with 50 μL of metformin. Row H and column 11 were reserved for metformin‐only and antibiotic‐only controls, respectively. Growth controls (wells A‐B, column 12) and sterility controls (wells C‐D, column 12) were also included. Following the incubation, MIC was determined for each well as the lowest concentration that showed no visible growth, verified using a spectrophotometer at 600 nm. The combination outcome was evaluated by calculating the FICI, defined as the sum of FICs for each tested drug. The FIC of metformin was calculated as the MIC of metformin in the combination divided by its MIC alone; similarly, the FIC of the antibiotic was calculated as the MIC of the antibiotic in the combination divided by its MIC alone. The interaction was interpreted as synergistic if (FICI ≤ 0.5), additive if (0.5 > FICI ≤ 1), indifferent if (1 < FICI ≤ 4), and antagonist if (FICI > 4) [36, 37]. All the combinational studies were performed in duplicate to ensure reproducibility.

### 2.7. Ethical Consideration

This study was approved by the Institutional Review Board (IRB) of An‐Najah National University (Ref: Mas. April 2024/22) and the Clinical Research Center (CRC) of An‐Najah National University Hospital (NNUH) (Ref: CRC_2024_0332). The research followed all relevant ethical guidelines and regulations and comes in compliance with the Helsinki Declaration.

### 2.8. Consent to Participate

Gastric biopsies from patients undergoing a routine gastroscopic procedure at An‐Najah National University Hospital (NNUH) were used to isolate *H. pylori.* Agreement to participate in the study and to provide a written consent was essential for study enrollment. Results of the three *H. pylori* clinical strains and their antibiotic profiles were used for guiding the treatment options.

## 3. Results

### 3.1. MIC and MBC of Antimicrobial Agents

The susceptibility of the two reference and the three clinical strains of *H. pylori* to six different antimicrobial agents was investigated using the broth microdilution assay to determine MIC values. The MIC and MBC results are summarized in Table 1. All the strains were susceptible to tetracycline and rifampicin. The reference strains showed susceptibility to all tested antibiotics except metronidazole, to which ATCC 43504 was resistant. Remarkably, all the clinical isolates were metronidazole‐resistant. The clinical isolate S1 is a multidrug‐resistant strain showing resistance to metronidazole, amoxicillin, clarithromycin, and levofloxacin. In contrast, the S3 isolate had a dual resistance to both metronidazole and clarithromycin. Overall, two out of the three clinical strains (S1 and S3) demonstrated resistance to clarithromycin.

**TABLE 1:** Minimum inhibitory concentration (MIC) and minimal bactericidal concentration (MBC) of the six antimicrobial agents tested against the 2 reference and the 3 clinical strains of *H. pylori* using the broth microdilution assay.

Antimicrobial agent	Average MIC ± SD μg/mL (MBC) μg/mL Susceptibility				
	Reference strains		Clinical strains		
	ATCC 43504	ATCC 700824	S1	S2	S3
MNZ	46.9 ± 18	0.61 ± 0.24	39.06 ± 15.6	39.06 ± 15.6	13.65 ± 3.9
	(62.5)	(1.9)	(62.5)	(62.5)	(31.25)
	R	S	R	R	R

AMX	0.02 ± 0.006	0.005 ± 0.001	0.78 ± 0	0.04 ± 0.01	0.098 ± 0
	(0.024)	(0.006)	(1.56)	(0.0975)	(0.098)
	S	S	R	S	S

CLA	0.068 ± 0.019	0.052 ± 0.02	5 ± 0	0.002 ± 0.0006	2.5 ± 0
	(0.078)	(0.065)	(5)	(0.078)	(2.5)
	S	S	R	S	R

TCN	0.11 ± 0.031	0.06 ± 0	0.11 ± 0.03	0.06 ± 0	0.03 ± 0.010
	(0.11)	(0.122)	(0.15)	(0.214)	(0.03)
	S	S	S	S	S

RIF	0.521 ± 0.18	0.521 ± 0.18	0.625 ± 0	0.521 ± 0.18	0.313 ± 0
	(1.67)	(2.08)	(0.625)	(1.25)	(0.313)
	S	S	S	S	S

LVF	0.24 ± 0.097	0.122 ± 0.04	21.88 ± 6.25	0.195 ± 0	0.39 ± 0
	(0.39)	(0.098)	(25)	(0.24)	(0.39)
	S	S	R	S	S

*Note:* MNZ: metronidazole, AMX: amoxicillin, CLA: clarithromycin, TCN: tetracycline, RIF: rifampicin, and LVX: levofloxacin. R: resistance and S: susceptible based on EUCAST breakpoints Version 16.0 [27]. Values represent mean ± standard deviation for the broth microdilution that is conducted in triplicate.

### 3.2. Metformin MIC and MBC

To investigate the antibacterial activity of metformin against multiple strains of *H. pylori,* MIC and MBC were determined using the broth microdilution method. As demonstrated in Table 2, metformin exhibited antibacterial activity against both the reference and clinical *H. pylori* strains. Metformin MICs ranged from 6.83 to 9.75 mM (1131–1615 μg/mL) across all strains tested, including MDR isolates. The MBC values matched the MIC values; the MBC/MIC ratio was 1, reflecting a bactericidal activity of metformin against *H. pylori*. In conclusion, these findings demonstrated metformin’s ability to inhibit various strains of *H. pylori*, regardless of the strain’s antibiotic resistance profile.

**TABLE 2:** In vitro minimal inhibitory concentrations (MICs) and bactericidal concentrations (MBCs) of metformin and MBC/MIC ratio against the two reference and the three clinical strains of *H. pylori* using the broth microdilution assay.

Isolates	Metformin MIC (mM)	Metformin MIC (μg/mL)	MBC (mM)	MBC/MIC ratio	Interaction
ATCC 43504	9.75 ± 3.9	1615 ± 646	9.75 ± 3.9	1	Bactericidal
ATCC 700824	9.75 ± 3.9	1615 ± 646	9.75 ± 3.9		
S1	7.81 ± 0	1293 ± 0	7.81 ± 0		
S2	6.83 ± 1.95	1131 ± 323	6.83 ± 1.95		
S3	7.81 ± 0	1293 ± 0	7.81 ± 0		

*Note:* MBC/MIC ratio is ≤ 4, the effect is considered bactericidal, and if the MBC/MIC ratio is > 4, the effect is defined as bacteriostatic.

### 3.3. Killing Kinetics

**FIGURE 1:**
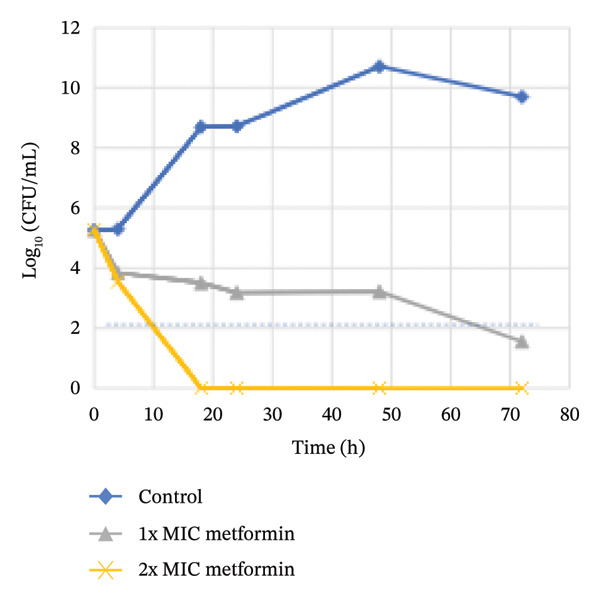
Killing kinetics curve for metformin on *H. pylori* reference strain ATCC 700824. The MIC value was 9.75 mM. The dotted line represents a 3‐log reduction in the number of bacteria compared to the initial inoculation (indicating bactericidal activity).

To evaluate the killing rate of *H. pylori* by different concentrations of metformin, a kinetic time‐kill study was performed against the reference strain ATCC 700824. As shown in Figure 1, control *H. pylori* cells produced an exponential growth phase; CFU/mL increased from a baseline of 5 log_10_ to > 10 log_10_ during the first 48 h of incubation. The results of challenging cells with 1‐2X MIC of metformin revealed a reduced bacterial load in a dose‐dependent manner. Exposure of *H. pylori* to 1X MIC of metformin caused a gradual decline reaching 3‐log_10_ reduction by < 72 h. Additionally, cellular count remained persistently low after an initial drop, indicating persistent but slower bactericidal activity (time‐dependent). Meanwhile, *H. pylori*‐treated cells with 2X MIC of metformin decreased the viability of *H. pylori* by ∼5 log10 during < 20 h; reflecting a rapid bactericidal activity, with the count dropped below the detection limit (0 log_10_) within 18 h. This effect was maintained during the 72 h period, confirming the rapid and complete bactericidal activity of metformin.

### 3.4. Synergy Testing of Metformin–Antibiotic Combinations

The in vitro interactions between metformin and the selected antibiotics were assessed using checkerboard broth microdilution assay across the same *H. pylori* strains tested previously. Results are shown in Table 3. The combination of metformin with each antibiotic led to a reduction in MIC values compared to when the agents were used individually. However, neither synergy nor antagonism was observed. FICI values for the combinations studied ranged from 0.52 to 1.56. Results between 0.52 and 1.0 indicated additive effects.

**TABLE 3:** Broth microdilution checkerboard results for metformin–antibiotic combinations against the two reference strains (ATCC 43504 and ATCC 700824) and the three clinical strains (S1, S2, and S3).

Antibiotic combined with metformin	Isolate identifier	FICI	Interpretation
MNZ	ATCC43504	0.99	Additive
	ATCC700824	1.13	Indifferent
	S1: (MDR)	0.81	Additive
	S2: (MNZ‐R)	0.87	Additive
	S3: (MNZ‐R, CLR‐R)	1.0	Additive

AMX	ATCC43504	1.56	Indifferent
	ATCC700824	0.52	Additive
	S1: (MDR)	1.09	Indifferent
	S2: (MNZ‐R)	0.76	Additive
	S3: (MNZ‐R, CLR‐R)	1.0	Additive

CLA	ATCC43504	1.02	Indifferent
	ATCC700824	1.51	Indifferent
	S1: (MDR)	1.13	Indifferent
	S2: (MNZ‐R)	1.0	Additive
	S3: (MNZ‐ R, CLR‐R)	0.75	Additive

TCN	ATCC43504	0.81	Additive
	ATCC700824	1.25	Indifferent
	S1: (MDR)	1.13	Indifferent
	S2: (MNZ‐R)	1.1	Indifferent
	S3: (MNZ‐R, CLR‐R)	0.83	Additive

LEV	ATCC43504	1.45	Indifferent
	ATCC700824	1.04	Indifferent
	S1: (MDR)	0.69	Additive
	S2: (MNZ‐R)	1.06	Indifferent
	S3: (MNZ‐R, CLR‐R)	0.63	Additive

RIF	ATCC43504	0.79	Additive
	ATCC700824	1.0	Additive
	S1: (MDR)	0.62	Additive
	S2: (MNZ‐R)	1.31	Indifferent
	S3: (MNZ‐R, CLR‐R)	1.03	Indifferent

*Note:* Combination outcome is considered synergy if the (FICI ≤ 0.5), additive if (0.5 > FICI ≤ 1), indifferent if (1< FICI ≤ 4), and antagonist if (FICI > 4). FICI represents the fractional inhibitory concentration index of both drugs calculated using the following equation, FICI = FIC for the antibiotic + FIC for metformin. MDR: multidrug‐resistant (S1 is an MDR isolate based on Table 1 results). R: resistant, MNZ: metronidazole, AMX: amoxicillin, CLA: clarithromycin, TCN: tetracycline, RIF: rifampicin, and LVX: levofloxacin.

Metformin–metronidazole combinations resulted in the most frequent additive interactions, observed in 4 out of 5 strains—all of which were resistant to metronidazole. An additive effect was also found in three out of five strains when metformin was paired with rifampicin or amoxicillin. Fewer additive interactions (2 out of 5 strains) were seen with combining metformin and levofloxacin, tetracycline, or clarithromycin. Notably, the metformin–levofloxacin combination produced the most significant reduction in levofloxacin concentration against a multidrug‐resistant *H. pylori* strain, with an FIC value of 0.19 (see Supporting Table 1: detailed FIC values). Collectively, the detected additive outcomes suggest potential benefits of using metformin in combination therapy aimed at overcoming antibiotic‐resistant *H. pylori* infections.

## 4. Discussion

*H. pylori* is one of the highly notable human pathogens; its adult global prevalence is close to 44% compared to 31% in children and adolescents between 2011 and 2022 [38]. The escalating level of antibiotic resistance and multidrug resistance (MDR) among *H. pylori* consequently limited the success of standard treatment protocols [39, 40]. In Palestine, recent findings have highlighted both high prevalence and an increased level of antibiotic resistance among *H. pylori* [41–44]. Thus, finding novel therapeutic strategies is crucial. One promising strategy is the use of non‐antibiotic agents as adjuvants to boost the efficacy of conventional antibiotics and help overcome resistance barriers [8].

Metformin is the most prescribed glucose‐lowering drug for T2DM [10, 45, 46]. Beyond its antidiabetic role, numerous studies have highlighted metformin’s antibacterial activity and its potential to act as an adjuvant to enhance the activity of the pre‐existing antibiotics against MDR pathogens. This antibacterial potential has been observed across numerous Gram‐positive and Gram‐negative bacteria [13, 14, 47–51]. However, its activity against *H. pylori* has not been fully explored. Therefore, this study aimed to investigate metformin’s activity against *H. pylori*. It also aimed to evaluate its synergistic potential when combined with six antibiotics commonly used in first‐ and second‐line treatment regimens.

In the current study, metformin exhibited MIC values of 9.75 mM (1615 μg/mL) against *H. pylori* reference strains and 6.83–7.81 mM (1131–1293 μg/mL) for the clinical strains. These findings suggest a potential antibacterial activity of metformin against *H. pylori,* including the MDR strains. This aligns with the findings of Courtois et al., who indicated a direct anti‐*H. pylori* effect of metformin using both in vivo and in vitro assays. Their disk diffusion results reflected antibacterial activity at concentrations of 0.5 and 1 M, while 0.1 M showed no effect. In the same report, values of the survival curve showed that 10 mM metformin significantly reduced *H. pylori* count in the treated cells [52]. Moreover, a previous study reported a direct effect for metformin in *H. pylori*‐infected mice, where oral administration of metformin successfully altered the composition of the gut microbiota [12]. However, our findings differ from the recently published report by Valadbeigi et al., who assessed the antibacterial activity for metformin against a clarithromycin‐resistant *H. pylori* clinical isolate. Their study reported an MIC value of > 500 μM, which is lower than the MIC values observed in our study [18]. These discrepancies may be due to multiple factors. While the two studies used the broth microdilution assay, differences in the assay’s protocol, including the inoculum density, media composition, incubation duration, and strain’s heterogeneity, could influence the outcomes [53]. Additionally, factors like the pH of metformin solution and the choice of solvent may further contribute to the observed differences in MIC values [54].

In our study, the MBC of metformin was found to be identical to its MIC, reflecting the bactericidal activity of metformin against the tested *H. pylori* strains. Similar killing potential was reported by Masadeh et al. against MRSA and *P. aeruginosa* [13]. Likewise, a bactericidal effect was observed in *Streptococcus suis* [55]. In contrast, Wu and his colleagues were unable to detect metformin’s bactericidal potential against *Enterococcus fecalis* [56], possibly due to their limited concentration range that did not exceed the MIC.

Further, our time‐killing curve results revealed metformin killing of *H. pylori* ATCC 700824 to be concentration‐dependent. Reflecting a potent bactericidal activity achieved with total eradication of *H. pylori* populations at 2X MIC within 18 h, this killing was maintained during the 72 h, confirming the absence of regrowth and possible inhibition of resistant mutants’ selection. The observed bactericidal activity may suggest a crucial mechanistic potential, targeting life‐threatening bacterial components, including the plasma membrane, the cell wall, DNA/RNA synthesis, or even protein synthesis [57]. A recent transcriptomic analysis uncovered metformin activity to actively reduce the expression of genes related to plasma membrane integrity and other metabolic genes among *E. coli* strains, contributing to its bactericidal activity [58]. Moreover, molecular docking analyses described multiple *H. pylori* target proteins with bactericidal activity, including enzymes in the shikimate pathway responsible for the production of essential aromatic amino acids and folate crucial for *H. pylori* survival, including shikimate kinase and chorismate synthase [59] and peptide deformylase, a key enzyme essential for the protein synthesis process [60]. However, the specific mechanism beneath the bactericidal effect of metformin against *H. pylori* needs to be elucidated. The ability of metformin to exert bactericidal effects against both antibiotic‐sensitive and MDR *H. pylori* strains could hold a therapeutic potential if approved by further analysis [61]. This finding is especially relevant if a synergistic bactericidal outcome resulted from its combination with antibiotics, leading to an enhanced killing at lower doses [62]. Multiple proposed mechanistic analyses revealed the synergy may be mediated by inhibition of efflux pumps among *S. aureus* when combined with beta‐lactam antibiotics [58], regulating its respective efflux gene, *tet(B)*, in *Pasteurella multocida* following doxycycline combination, thereby increasing its membrane permeability [63]. On the other hand, the bactericidal activity of metformin can be used in the design of novel surface agents for endoscopy rooms and gastroscopic devices [64].

The interaction between metformin and different antibiotics was determined via FICI analysis. The FICI values ranged between 0.56 and 1.0, reflecting an additive effect when metformin was combined with each of the six tested antibiotics (amoxicillin, clarithromycin, tetracycline, metronidazole, levofloxacin, and rifampicin). These additive effects were not related to the resistance pattern of bacterial strains, as similar outcomes were recorded for both the reference strains and the clinical isolates, including the MDR (S1). The additive activity of combining metformin with metronidazole was detected in four out of five strains, while the combinations with the other antibiotics showed additive effects in three and two out of five strains. The remaining FICI results for isolates that did not show additive interactions were 1> FICI ≤ 1.56, indicating neutral interaction; no evidence of antagonism or synergy was detected. To date, only one recent study has investigated the combination of metformin and amoxicillin against *H. pylori*. In contrast to our findings, the study reported a strong synergistic activity for the combination with FICI 0.34. Thus, suggesting metformin’s adjuvant role in enhancing amoxicillin efficacy against *H. pylori* [18]. In our study, FICI results for the same combination were 0.52 and 0.76, indicating additive effects. Differences in FICI findings across studies could be due to multiple factors, including strains’ heterogeneity, diverse resistance mechanisms, and variations in experimental protocols such as cellular density, media composition, and incubation conditions. Moreover, the concentration of metformin and amoxicillin used in the combination and the pH of the working solution may also contribute to such discrepancies in the FICI values [65]. On the other hand, multiple previous investigations reported metformin’s adjuvant activity when paired with doxycycline. In one of those studies, metformin was identified as a novel enhancer of tetracycline activity against MDR Gram‐positive and Gram‐negative bacteria harboring the *tet(A*) resistance gene. The study detected synergistic interactions with all investigated tetracyclines except tigecycline. However, an additive effect was noticed among tetracycline‐susceptible strains [47]. This could partly account for the additive interactions noted in our study between metformin and tetracycline, as none of the tested *H. pylori* strains exhibited resistance to tetracycline. Similarly, Masadah et al. reported a synergistic outcome for combining metformin with antibiotics, including doxycycline and levofloxacin, when tested against MRSA and *P. aeruginosa* [13]. The synergy of the metformin–doxycycline combination was also reported by Hossain et al. using in vitro, in vivo, and in silico models against *Bacillus cereus* and *Shigella boydii* [49]. In related studies, researchers investigated the mechanism behind this synergy, revealing that metformin could enhance the intracellular concentration of the antibiotic, modulate immune responses, and inhibit bacterial efflux pump activity [47, 50, 51, 63]. In a similar context, combining metformin with minocycline has been shown to destroy the outer membrane integrity of *A. baumannii* with increased membrane potential [14].

Although the synergy interactions have been well documented against MDR‐Gram‐negative and Gram‐positive isolates, these findings may not be directly applicable to *H. pylori*. Therefore, further investigations are highly required to validate the outcomes of these combinations against *H. pylori*. The checkerboard findings reported no antagonistic activity for the studied combinations; in contrast to our data, a previous report showed an antagonistic combinational outcome with levofloxacin against *K. pneumoniae* [52]. The team attributed the antagonism to the proposed antioxidant activity of metformin that reduced the oxidative stress‐mediated bactericidal potential of fluoroquinolones [51].

The exact mechanism underlying the antibacterial activity of metformin remains incompletely understood. However, existing literature has proposed several hypotheses, including its potential activation of AMPK, possible production of mitochondrial ROS against *M. tuberculosis* [19], and its antivirulence properties, including biofilm inhibition in Gram‐negative bacteria like *P. aeruginosa, K*. *pneumoniae,* and *E. coli* [20, 21, 58]. Additionally, Shafik et al. reported a remarkable role for metformin in decreasing urease production from *K. pneumoniae* [21]. For *H. pylori*, a previous report demonstrated metformin’s likelihood of downregulating the expression of the *cagA* gene [18]. In the same context, metformin consumption in patients with *H. pylori* infection has also been shown to suppress the PTEN promoter methylation, in addition to its role in reducing ROS and subsequent inhibition of matrix metalloproteinase 10, potentially lowering the risk of gastritis and gastric cancer [66, 67]. Future investigations focused on the mechanistic evaluation of metformin anti‐*H. pylori* activity are crucial. Our future work will focus on both phenotypic and genotypic assessment for the activity of metformin on *H*. *pylori* biofilm formation, urease production, and other virulence factors, including the *cagA* gene.‬‬‬‬‬‬‬‬‬‬‬‬‬‬‬‬‬‬‬‬‬‬‬‬

## 5. Strengths and Limitations

One of the key strengths of this study is the use of clinical and reference strains in the evaluation of metformin’s antibacterial activity against *H. pylori*, including those antibiotic‐susceptible, resistant, and MDR. Additionally, implementing a checkerboard assay gave a systematic framework for quantifying metformin–antibiotic combinations. Finally, the time‐kill assay provided a dynamic assessment for metformin bactericidal activity.

However, the study has several limitations; the small number of clinical isolates restricts the ability to generalize the results, particularly considering the substantial genetic diversity present within *H. pylori* populations. Therefore, future research should incorporate a larger set of clinical isolates along with genotypic analysis of resistance markers such as mutations in *23S rRNA*, *gyrA*, and *rdxA* genes to strengthen the current findings and to include relevant statistical analysis. Another limitation is the absence of any tetracycline‐resistant strains among the isolates tested. Furthermore, validating the anti‐*H. pylori* effects of metformin through in vivo studies, along with a thorough evaluation of its potential toxicity, is essential. Finally, metformin’s concentrations (MIC) used in the study, ranging between 6.83 and 9.75 mM (1131–1615 μg/mL), exceed the typical systemic levels and may not directly reflect clinical applicability. However, its concentrations in the GI system can be 10 to 300 times higher than in plasma; previous studies have reported gut lumen concentrations range from 10 to 50 mM due to its low bioavailability and partial absorption [68]. Hence, these concentrations are still relevant for exploring potential local effects in the gastrointestinal tract and for identifying possible synergistic interactions in the vitro settings.

## 6. Conclusion

To the best of our knowledge, this is the first study to investigate the potential synergy of metformin with six common conventional antibiotics that are used in the first‐ and second‐line treatments of *H. pylori* infections. It is important to highlight that in vitro, metformin demonstrates a direct but weak anti‐*H. pylori* potential with bactericidal activity, and it has an additive effect when combined with all the tested antibiotics, and it has no antagonistic action among the tested isolates. Among diabetic patients, metformin may provide the added advantage of both glycemic control and potential support in combating infections. However, further studies are required to explore its efficacy and safety.

**Nomenclature**
*H. pylori*
: *Helicobacter pylori*
MDR: Multidrug‐resistant
ADA: American Diabetes Association
WHO: World Health Organization
DM: Diabetes mellitus
AMPK: Adenosine monophosphate‐activated protein kinase
ROS: Reactive oxygen species
ATCC: American‐type culture collection
DMSO: Dimethyl sulfoxide
MIC: Minimal inhibitory concentration
MBC: Minimal bactericidal concentration
FICI: Fractional inhibitory concentration index
NNUH: An‐Najah National University Hospital
BHI: Brain‐heart infusion
FBS: Fetal bovine serum
MHB: Muller–Hinton broth
EUCAST: European Committee on Antimicrobial Susceptibility Testing
MNZ: Metronidazole
TCN: Tetracycline
LEV: Levofloxacin
RIF: Rifampicin
AMX: Amoxicillin
CLA: Clarithromycin
S: Susceptible
R: Resistant
CRC: Clinical Research Center

## Author Contributions

Walaa Hashem Rajab conceptualized and designed the study, conducted the literature search, performed lab work, and wrote the manuscript. Nidal Jaradat conceptualized the study and resources. Qusay Abdo conceptualized the study and sample collection. Nour Aljouda supervised the lab work and revised the manuscript. Mohammad Qadi conceptualized and designed the study, conducted the literature search, supervised the lab work, and revised and wrote the manuscript. All authors discussed the results and contributed to the final manuscript.

## Funding

No funding was received for this study, but it was supported by the An‐Najah National University as part of a doctoral degree project.

## Ethics Statement

This study was approved by the Institutional Review Board (IRB) of the An‐Najah National University (Ref: Mas. April 2024/22) and the Clinical Research Center of the An‐Najah National University Hospital (NNUH) (Ref: CRC_2024_0332). The research followed all relevant ethical guidelines and regulations and comes in compliance with the Helsinki Declaration.

## Consent

Gastric biopsies from patients undergoing routine gastroscopic procedure at the An‐Najah National University Hospital (NNUH) were used to isolate *H. pylori*. Agreement to participate in the study and to provide a written consent was essential for study enrollment. Results of the three *H. pylori* clinical strains and their antibiotic profile were used for guiding the treatment options.

Every participant was clearly informed about the purpose of the study before participating, and their consent was obtained with their signature on the form.

## Conflicts of Interest

The authors declare no conflicts of interest.

## Supporting Information

Additional supporting information can be found online in the Supporting Information section.

## Supporting information


**Supporting Information** Supporting Table 1: Broth microdilution checkerboard results for metformin–antibiotic combinations against the two reference strains (*Helicobacter pylori* ATCC 43504 and ATCC 700824) and the three clinical strains (S1, S2, and S3). Detailed fractional inhibitory concentration (FIC) values are provided in Supporting Table 1.

## Data Availability

The data used to support the findings of this study are included in the manuscript. Additional details regarding data availability, including access to the raw data analyzed in this study, can be obtained by contacting the corresponding author (Mohammad Qadi; m.qadi@najah.edu).
