# Supplementary material for: Antimicrobial and Synergistic Activity of Metformin Against Helicobacter pylori
Source: Can J Infect Dis Med Microbiol. 2026 May 12;2026:3542284. doi: 10.1155/cjid/3542284 (PMC13161997; doi:10.1155/cjid/3542284)
Supplement: Supplementary file 1 — Supporting Information Supporting Table 1: Broth microdilution checkerboard results for metformin–antibiotic combinations against the two reference strains (Helicobacter pylori ATCC 43504 and ATCC 700824) and the three clinical strains (S1, S2, and S3). Detailed fractional inhibitory concentration (FIC) values are provided in Supporting Table 1. [file CJID-2026-3542284-s001.docx]

**Supplementary Table 1: Broth microdilution checkerboard results for Metformin-antibiotic combinations against the two reference strains (ATCC 43504, ATCC 700824) and the three clinical strains (S1, S2 and S3)**

| Antibiotic  combined with Metformin | Isolate  Identifier | | FIC for antibiotic | FIC for metformin | FICI | Interpretation |
| --- | --- | --- | --- | --- | --- | --- |
| MNZ | ATCC43504 | 0.63 | | 0.36 | 0.99 | Additive |
|  | ATCC700824 | 0.125 | | 1.0 | 1.13 | Indifferent |
|  | S1: (MDR) | 0.31 | | 0.5 | 0.81 | Additive |
|  | S2: (MNZ-R) | 0.37 | | 0.5 | 0.87 | Additive |
|  | S3: (MNZ-R, CLR-R) | 0.5 | | 0.5 | 1.0 | Additive |
|  |  |  | |  |  |  |
| AMX | ATCC43504 | 1.5 | | 0.06 | 1.56 | Indifferent |
|  | ATCC700824 | 0.4 | | 0.12 | 0.52 | Additive |
|  | S1: (MDR) | 1.0 | | 0.09 | 1.09 | Indifferent |
|  | S2: (MNZ-R) | 0.73 | | 0.03 | 0.76 | Additive |
|  | S3: (MNZ-R, CLR-R) | 0.49 | | 0.51 | 1.0 | Additive |
|  |  |  | |  |  |  |
| CLA | ATCC43504 | 1.0 | | 0.02 | 1.02 | Indifferent |
|  | ATCC700824 | 1.0 | | 0.51 | 1.51 | Indifferent |
|  | S1: (MDR) | 0.38 | | 0.75 | 1.13 | Indifferent |
|  | S2: (MNZ-R) | 0.75 | | 0.25 | 1.0 | Additive |
|  | S3: (MNZ- R, CLR-R) | 0.25 | | 0.5 | 0.75 | Additive |
|  |  |  | |  |  |  |
| TCN | ATCC43504 | 0.75 | | 0.06 | 0.81 | Additive |
|  | ATCC700824 | 0.75 | | 0.5 | 1.25 | Indifferent |
|  | S1: (MDR) | 0.13 | | 1.0 | 1.13 | Indifferent |
|  | S2: (MNZ-R) | 1.0 | | 0.1 | 1.1 | Indifferent |
|  | S3: (MNZ-R, CLR-R) | 0.75 | | 0.08 | 0.83 | Additive |
|  |  |  | |  |  |  |
| LEV | ATCC43504 | 0.70 | | 0.75 | 1.45 | Indifferent |
|  | ATCC700824 | 1.0 | | 0.04 | 1.04 | Indifferent |
|  | S1: (MDR) | 0.19 | | 0.5 | 0.69 | Additive |
|  | S2: (MNZ-R) | 1.0 | | 0.06 | 1.06 | Indifferent |
|  | S3: (MNZ-R, CLR-R) | 0.50 | | 0.13 | 0.63 | Additive |
| RIF | ATCC43504 | 0.53 | | 0.26 | 0.79 | Additive |
|  | ATCC700824 | 0.75 | | 0.25 | 1.0 | Additive |
|  | S1: (MDR) | 0.5 | | 0.12 | 0.62 | Additive |
|  | S2: (MNZ-R) | 1.24 | | 0.07 | 1.31 | Indifferent |
|  | S3: (MNZ-R, CLR-R) | 1.0 | | 0.03 | 1.03 | Indifferent |

Combination outcome is considered synergy if the (FICI ≤0.5), additive if (0.5> FICI ≤ 1), indifferent if (1< FICI ≤ 4) and antagonist if (FICI > 4). FICI represents the fractional inhibitory concentration index of both drugs calculated using the following equation, FICI = FIC for the antibiotic + FIC for metformin

Abbreviations: MDR: Multidrug resistant (S1 is a MDR isolate based on Table 1 results). R: Resistant MNZ: Metronidazole, AMX: Amoxicillin, CLA: Clarithromycin, TCN: Tetracycline, RIF: Rifampicin and LVX: Levofloxacin.
